# Perception and Acceptance of People with Disabilities by Employers and Co-Workers

**DOI:** 10.3390/ijerph18105278

**Published:** 2021-05-16

**Authors:** Alicja Grześkowiak, Urszula Załuska, Cyprian Kozyra, Dorota Kwiatkowska-Ciotucha

**Affiliations:** 1 Department of Econometrics and Operational Research, Wroclaw University of Economics and Business, 53-345 Wrocław, Poland; alicja.grzeskowiak@ue.wroc.pl; 2 Department of Logistics, Wroclaw University of Economics and Business, 53-345 Wrocław, Poland; urszula.zaluska@ue.wroc.pl; 3 Department of Statistics, Wroclaw University of Economics and Business, 53-345 Wrocław, Poland; cyprian.kozyra@ue.wroc.pl

**Keywords:** disability, WHO ADS scale, inclusive employment, acceptance of types of disability, confirmatory factor analysis, measurement invariance, structural equation modelling

## Abstract

The perception of people with disabilities is crucial for their full inclusion and in order that they might stay economically active. The measurement tools used should be resistant to the demographic or professional characteristics of the research participants. The article attempts to test this resistance for one of the most popular tools measuring the perception of people with disabilities in everyday life—the Attitudes to Disability Scale (ADS) test developed by the WHOQOL Group. Another issue raised in the article is the acceptance of people with various types of disabilities in terms of their possible employment. We checked the differentiation of acceptance among employers from different countries. This article uses representative samples of respondents from two studies—the CATI research (2019) on samples of Polish employers and co-workers, and the CAWI research (2021) on samples of employers from Poland and Finland. The analysis methods used included confirmatory factor analysis, nested models and nonparametric analysis of variance. The research confirmed the resistance of the ADS scale to respondents’ characteristics, and found no differences for nested models constructed for groups based on categorical variables characterizing the respondents. As for acceptance of various types of disability in the workplace, significant differences were found in the statements of employers from Poland and Finland.

## 1. Introduction

Increasing life expectancy, aging of societies and technological progress, especially in the field of medicine, make people with disabilities (PwD) constitute an ever larger group. According to data gathered by Eurostat, almost 25% of European Union citizens (24.7%) aged 16 and more are people with some or severe limitations [1,2]. In absolute figures, this is at least 110 million people. According to global estimates, the number of people with disabilities exceeds one billion, or 15% of the world’s population [3,4,5]. From the legal point of view, these people are ensured normal functioning in society and equal access to workplaces. This has been stated in the most important international document guaranteeing full social inclusion, namely the Convention on the Rights of Persons with Disabilities adopted by the United Nations in 2006 [6]. However, it is necessary to remember that equal treatment of people with disabilities is not a simple derivative of international law ratified by many countries. According to official data, in most European and world countries the employment rate for this group of people is much lower than for the remaining part of society and causes or consequences of this phenomenon are analysed in many studies [7,8,9,10,11,12,13,14]. The reason for this is, among others, the unfavorable social perception of people with disabilities as a consequence of fear and stigma, the source of which lies in insufficient awareness of the specificity of various types of disability. As shown by the results of research conducted by various teams, the main source of knowledge about disability is previous experience in cooperation with PwD [15,16,17]. It can be observed that employers who have already employed PwD are more likely to decide to hire people from this group. Similarly, co-workers who have people with disabilities in their families or among friends are more open and more willing to accept them in the workplace [18]. However, in the case of able-bodied people without prior personal contact with PwD, the key role in gaining knowledge about disability is played by popular culture texts in the press, radio, television, books etc. Unfortunately, the message that they convey does not show PwD in a favourable light [18,19]. Disability is presented as a constant struggle with adversities/handicap (with focus on “heroism”) or something that excludes people from society and makes them dependent on others (“weakness”). The image of a person with disability living a “normal” life, without prejudice or unnecessary overtones, is rare to see. Depicting PwD in such a way fosters social dislike towards this group, and it is the positive attitude of employers and able-bodied co-workers that is essential for a successful adaptation process for people with disabilities in the workplace [20,21].

In the context of successful professional activation, it is crucial to develop a universal and reliable tool to measure the perception of people with disabilities, both in everyday life and in the workplace. A review of those that are available shows that, although many instruments are already used, most of them have certain limitations, e.g., with regard to the dedicated target group [15], and require further verification to broaden their scope of use. In the area of measurement tools, the work of the team led by M. Power (The World Health Organization Quality of Life Group—WHOQOL Group) on the development of the Attitudes to Disability Scale (ADS) test is particularly important [22,23,24]. The ADS questionnaire asks about the respondent’s opinion on disability and disabled people in general. This tool contains 16 statements relating to the perception of the functioning of people with disabilities in society (detailed list of ADS items can be found in [22]). They have been grouped into four main fields (factors): Inclusion (items 1–4), Discrimination (items 5–8), Gains (items 9–12), Prospects (items 13–16). Respondents express their opinions using a 5-point Likert scale, where “1” means “I completely disagree”, whereas “5” means “I completely agree”. The authors of the ADS scale obtained good results in terms of measurement reliability in groups of people who experienced disability, i.e., people with disabilities and people directly related to them. In 2020, the authors of the article, inspired by the results obtained by the WHOQOL Group, as part of international research on representative samples of respondents from eight European countries, also confirmed the possibility of using this tool to measure attitudes of the general public, and not only among people with experience of disability [25]. The aim of the research was to test the resistance of this tool to the demographic and professional characteristics of the respondents. It should be noted, however, that the ADS scale measures only a declarative approach to the perception of disability, whereas differences between declarations and actual activity are usually significant, and not in favor of openness towards people with disabilities [26]. Therefore, we decided to check the degree of acceptance of people with various types of disabilities in the workplace. In this study, we were interested in the degree of acceptance of various types of disability and the existence of possible differences between employers from two countries of the European Union: Poland and Finland. We decided to use questions from the proprietary questionnaire presented in Table A1 in Appendix A. Poland and Finland were selected on purpose due to different cultural determinants resulting in different social atmospheres and approaches to disability in the workplace. The choice was also influenced by various welfare state regimes existing in both countries.

Eventually, three research questions were formulated:

The first research question: Is the WHO measurement tool (the ADS scale) developed by the WHOQOL Group [22] resistant to the demographic and professional characteristics of the respondents? In other words: Is the ADS scale the right tool to measure attitudes towards people with disability?

The second research question: Does openness towards people with disabilities (PwD) depend on the type of disability? In other words: Do people who declare openness towards e.g., people with reduced mobility, also declare openness towards other types of disability?

The third research question: Does the place where the company operates (country) differentiate the degree of acceptance of people with various types of disabilities in terms of their possible employment? In other words: Are the opinions of employers from different countries on the acceptance of, and willingness to employ, people with various types of disability similar?

## 2. Materials and Methods

### 2.1. Research Methods

In this article we used the results of two studies. The first one was carried out using the CATI method (Computer-Assisted Telephone Interview) in 2019 on random and quota samples of Polish employers and co-workers. Employers were understood as people responsible and jointly responsible for hiring employees, and co-workers as those who did not have such influence. The group of respondents consisted of people working in companies employing at least 5 people. As for employers, the sample was controlled due to the size of entity, and in the case of co-workers it reflected the structure of the working population in Poland in terms of age and gender. According to the assumptions, the sample size was at least 1000 people, including 300 employers and 700 co-workers. The research was carried out using a proprietary questionnaire, and the average interview duration was 15 min. Its main objective was to define attitudes and behaviors towards employing people with disabilities and to evaluate various aspects of the perception of PwD. The questions asked to the respondents concerned, among other topics, general perception of people with disabilities (using the ADS WHOQOL Group [22] scale) and perception of them in the workplace. The second piece of research was conducted using the CAWI method (Computer-Assisted Web Interview) in 2021 on samples of employers from Poland and Finland. According to the assumptions, the minimum sample from each country was 200 people and it was controlled due by size of entity. The research covered respondents from companies employing at least five people. They were recruited mainly from online panels available in a given country as well as company databases. The interview lasted about 12 min and covered two areas—preparing employees for current and future challenges, and openness towards employing PwD. In the section dedicated to PwD, the respondents were asked, among other questions, about the degree of acceptance of people with various types of disabilities in the workplace by co-workers from a given country and willingness to employ such people in their company.

### 2.2. Research Sample

During the CATI research a total of 1005 full interviews were carried out, including 301 in the group of employers and 704 among co-workers. The structure of the research sample in terms of selected demographic and professional characteristics of the respondents can be found in Table 1. We took into account those characteristics that were used in the analyses presented in the article. Due to the partial differentiation of information about the respondents’ characteristics in the research covering employers and co-workers, the analyses were carried out in three different cross-sections: for the entire research sample, for the sample of employers and for the sample including co-workers. In the case of the general sample, the differentiating variables were the character of one’s work (decision-maker/non-decision maker) and gender. In the group of employers, we took into account the basic type of company’s activity and its size, along with age in the group of co-workers. For the purpose of the research, we distinguished three age groups: young (18–34 years old), middle-aged (35–49 years old) and mature (50+).

People who participated in the research constituted a diverse group in terms of employment and experience in contact/cooperation with people with disabilities. During the research implementation period, in about half of the companies represented by employers, there were already some workers with disabilities, whereas other entities had employed them in the past. Among the remaining companies, there were both those where the employment of people with disabilities was considered and those where it was never taken into account. More than half of the co-workers stated that they had no contact with people with disabilities in their current workplace. Half of the respondents evaluated their knowledge about PwD as average, one third as good or very good, whereas the rest stated that they did not know anything about this topic. Co-workers also commented on their personal experience with people with disabilities. According to what they stated, 15% had no such experience. As far as other respondents are concerned, the most frequently mentioned relationships/situations in which they had gained some experience with PwD were those taking place in the circle of family and friends.

As part of the CAWI research, 415 complete questionnaires were collected from employers—215 from Poland and 200 from Finland. They represented companies of various sizes operating in various sectors of the economy, and on average they were 38 years old. As far as gender is concerned, the majority of respondents were men. We observed significant differences between countries, as the Polish sample was dominated by women, and the Finnish sample by men. The average percentage of companies employing people with disabilities was 31.8, with a significantly lower number of companies employing such workers in Poland than in Finland. More than one-third of the respondents described their knowledge about disability as good or very good. The characteristics of the research sample in terms of the analyzed features are presented in Table 2.

### 2.3. Methods of Data Analysis

In order to assess the validity of the ADS scale based on the data collected in the CATI research carried out among employees and employers in Poland, both as a whole and in terms of its invariance in relation to respondents’ demographic and professional characteristics, we used confirmatory factor analysis (CFA) [27]. To evaluate accuracy, we verified the model containing four latent variables representing the main dimensions of the ADS scale, and each of these was reflected in four questions that we asked to the respondents. The model was estimated on the basis of the entire sample, whereas the scale was evaluated through the prism of the obtained factor loadings, relationships between factors and using goodness-of-fit statistics: CMIN/DF (minimum discrepancy), RMSEA (root mean square error of approximation), GFI (goodness of fit index), AGFI (adjusted goodness of fit index), CFI (comparative fit index) and IFI (incremental fit index) [28]. Next, we performed CFA with a group division according to demographic and professional characteristics using nested models, and compared models containing parameters estimated for particular groups (configural model) with models taking into account constraints. Three types of constraints were considered: (1) relating to measurement weights, that is, formulated as equality of factor loadings in all groups, (2) relating to structural covariances, that is, taking into account the equality of factor loadings of variance and covariance between them in all groups, (3) defined as measurement residuals, that is, assuming that all parameters are constant across the groups [29] (pp. 383–384). It should be noted that the constraints of type (3) are very strong, therefore the results concerning (1) and (2) were mainly interpreted. We compared the results for models with constraints assuming that the unconstraint model is correct. As for the conclusion, we performed this using the classic approach based on the chi-square difference test, in which the test statistic is the difference between the value of the chi-square statistic for the configural model (no assumptions about equality of parameters) and the value of the chi-square statistic for a given variant of the model with imposed constraints [30] (p. 221). A high discrepancy of these statistics results in a low *p* value and is evidence of non-invariance, that is, differences in the measurement model occurring between the groups.

The differentiation in the degree of acceptance of people with various types of disabilities in terms of their possible employment was analysed using [31] nonparametric Mann-Whitney U test for two independent groups, nonparametric Friedman ANOVA test for one-way repeated measures analysis of variance, chi-square test of independence in a two-way table, exploratory factor analysis, reliability analysis with Cronbach’s alpha coefficient and [32] structural equation models, whose fit was assessed analogously to the CFA models.

In order to carry out statistical calculations and modelling we used statistical packages such as Statistica 12.5 [33] and IBM SPSS 25.0 (IBM Corp., Armonk, NY, USA), including statistical package SPSS AMOS [29] and MS Excel 2019 (Microsoft Corp., Redmond, WA, USA).

## 3. Results

### 3.1. Evaluation of the Measurement Validity by Confirmatory Factor Analysis—Whole Sample

Based on the results of research carried out on a sample of employees and employers using the Attitudes to Disability Scale (ADS) WHOQOL Group [22], a confirmatory factor analysis was performed to evaluate the measurement model. The model parameters were estimated from the data representing answers of both employers and co-workers, and according to the concept of the creators of the ADS scale, we used the structure consisting of four factors reflecting latent dimensions: Inclusion, Discrimination, Gains and Prospects. Each factor was represented by four observable variables, and in the construction of the model it was assumed that the factors were correlated with each other. We used two methods of estimation: maximum likelihood (ML) and asymptotically distribution free (ADF). Their selection was conditioned, on the one hand, by the universal use of (ML), and, on the other hand, by a set of weaker and more realistic assumptions in relation to the research material obtained from the questionnaires.

**Figure 1:**
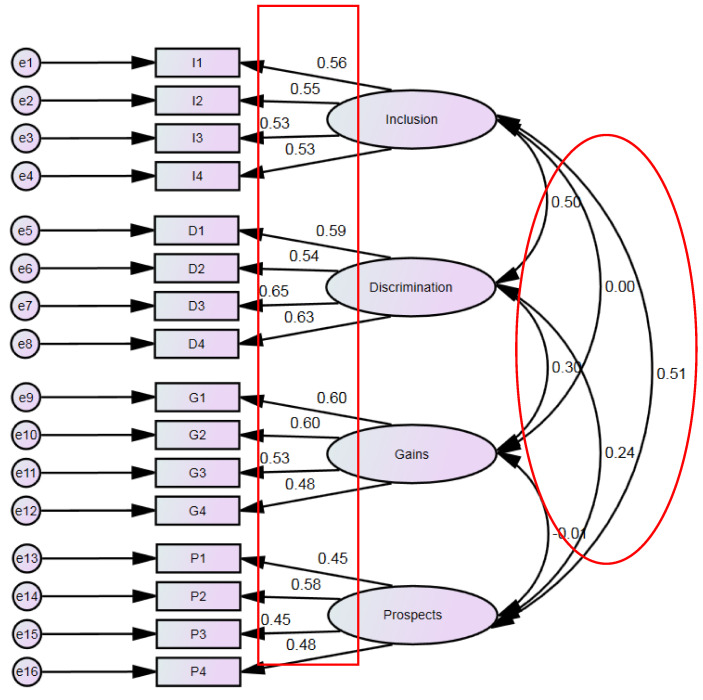
Results of the confirmatory factor analysis model—standardized estimates (ML method). Variable content—see [22].

The measurement model, standardized factor loadings estimated with the maximum likelihood method and the correlations between the factors are presented in Figure 1. For comparative purposes, the parameter estimates obtained with ML and ADF methods are shown in Table 3.

In the model with parameters estimated using ML, all factor loadings are characterized by high statistical significance (*p* < 0.001). Most of them exceed the value of 0.5, and in the cases below this threshold the deviations are small—the lowest value obtained is 0.453 (for P1 and P3). The highest values of loadings occurred for items that create the latent Discrimination variable, that is, D3 (0.648) and D4 (0.632). High statistical significance can also be noted when using ADF as an estimation method, although there was a greater variation in the values of loadings than when using ML—the lowest value was 0.279 (P3), whereas the highest was 0.706 (I1). The ground for using ADF was its non-parametric nature, but it should be noted that it requires large samples. This might be the reason for discrepancies in the results of ML estimation, which leads to further interpretation based mainly on the results of ML, especially in the case of division into groups according to categorical variables. In this model we obtained both significant and insignificant correlations between latent variables. The results of both estimation methods indicate that the Inclusion and Gains as well as Prospects and Gains variables are not correlated with each other. For the remaining pairs, significant positive correlations were found, although of not very high intensity—from 0.144 (Discrimination and Prospects, ADF) to 0.503 (Inclusion and Discrimination, ML).

In Table 4 we present the model quality measures recommended in the literature, that is, chi-square statistics and goodness-of-fit statistics for the four-factor model, including CMIN/DF (minimum discrepancy), RMSEA (root mean square error of approximation), GFI (goodness of fit index), AGFI (adjusted goodness of fit index), and IFI (incremental fit index). The chi-square statistic is 320.123 and is significant. This can be interpreted as a large discrepancy, an undesirable phenomenon, but due to the large sample size, the interpretation of this measure is highly problematic, as indicated, among others, by Hair et al. [34]. Therefore, it is better to use other descriptive indicators. One of the key indicators is RMSEA, which indicates the overall fit model.

Its value at the level of 0.048 indicates the acceptable error of approximation defined by Browne and Cudeck [35] as 0.08. Acceptable model fit is confirmed by absolute measures of GFI and AGFI fit exceeding 0.9, and relative measures determined in relation to the independence model, i.e., CFI and IFI, also above 0.9 [36,37]. The elements of the factor structure and measures obtained on the basis of the ML estimates allow a conclusion of the acceptance of the measurement model for the whole sample.

### 3.2. Evaluation of Multi-Group Measurement and Structural Invariance

The research participants did not create a homogeneous group. Firstly, the answers were given by both employers and (potential) co-workers of PwD. Secondly, the group of employers consisted of representatives of various sectors of the economy and enterprises of different size. Gender was also a potentially differentiating feature. Thirdly, in the group of employees the main demographic characteristics were gender and age. In order to assess whether there was any differentiation according to the above-mentioned characteristics, we relied on models that took into account the division into groups according to these criteria, using the nested model approach. We compared models containing the parameters estimated for specific groups (configural model) with those containing constraints (called measurement weights, structural covariances and measurement residuals). The results for nested models constructed for groups created according to categorical variables characterizing the respondents are presented in Table 5. We analysed the models on the basis of the whole sample—we examined the division into groups by employee/employer and gender, models based on data concerning employers. We also analysed the division into groups by economy sectors and the size of the enterprise, as well as by models based on data concerning co-workers—we examined group division according to age.

The results in Table 5 concerning the nested models for the entire sample show that there are no significant differences in the values of factor loadings and variance and covariance between them in the case of comparisons concerning gender and role played in the workplace (employer/employee), since the chi-square difference test score for measurement weights and structural covariances is characterized by high *p* values. Tests carried out with the division into the characteristics describing employers (sector, company’s size) gave results with quite varied difference statistics. While high *p* values in the case of company size (0.922 and 0.970) clearly indicate the similarity of parameters in the groups, *p* values for belonging to the sector (0.011 and 0.035) can be interpreted differently depending on the adopted significance level. Because of this, conclusions are not unambiguous. The results for the division into groups by age in the analysis of employees prove that there are no clear differences in factor loadings. At the same time, there is no equality for structural covariances. Summarizing the results for nested models, it is possible to conclude that the measurement model is invariant due to almost all the analysed characteristics, which means that the scale developed by the WHOQOL Group is an appropriate tool to measure attitudes towards people with disabilities.

### 3.3. Evaluation of Openness towards People with Various Types of Disability

Attitudes towards various types of disability in the workplace were analysed in the research conducted among employers from Poland and Finland using sets of questions about the acceptance of PwD in the workplace by co-workers and the willingness of employers to employ them. In both aspects, the questions concerned four types of disability (see Table A1 in Appendix A): 1. physical (N5_1, N6_1), 2. vision (N5_2, N6_2), 3. auditory disability with communication problems (N5_3, N6_3) and 4. cognitive disability, including intellectual and mental disability (N5_4, N6_4). The evaluations concerning specific types of disability were strongly correlated with each other, presented in Table 6 and Table 7. This means that the four questions, regardless of the analysed type of disability, measure one characteristic: overall perceived acceptance or general willingness to employ PwD.

**Figure 2:**
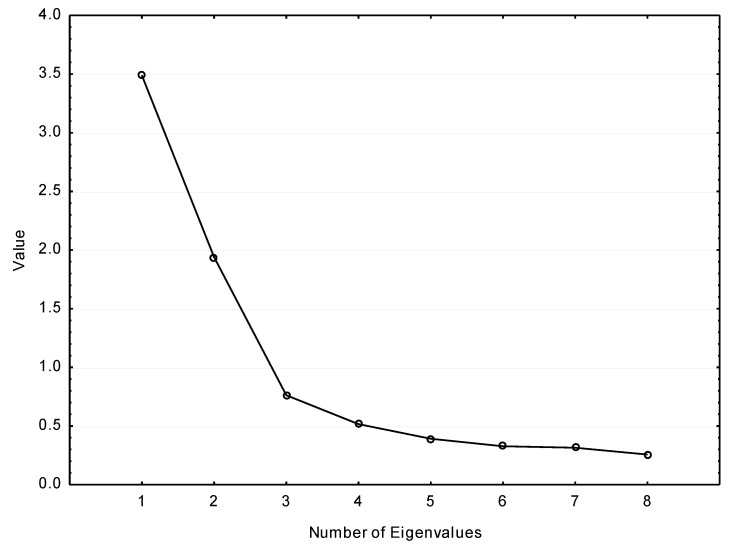
Plot of eigenvalues for joint items sets assessing PwD types.

The measurement of the dependency between questions from the sets about acceptance and willingness to hire PwD indicated much lower values of correlation coefficients, although significantly higher than zero. In the factor analysis performed with the method of principal components, we managed to distinguish two factors (presented in Figure 2), where only two eigenvalues are greater than 1. The factor loadings after varimax rotation are presented in Table 8.

The obtained results prompt the measurement of both attitudes towards PwD in the workplace using summative scales (Cronbach’s alpha equal to 0.868 for acceptance of PwD and 0.806 for willingness to employ PwD). Relationship between both constructs can be modelled as influence of perceived acceptance among co-workers on willingness to employ PwD by employer. Results of the structural equation model estimated by means of maximum likelihood (ML) methods with standardization are presented in Table 9.

Assessing the fit of the model in Table 9, it can be concluded that it should be improved, because some measures have worse values than recommended in the literature, but it is sufficient good to assess the regression coefficient between Acceptance and Willingness latent variables equal to 0.323 as significantly positive, however, showing only moderate impact of perceived acceptance PwD among coworkers on willingness to employ PwD by employer. Thus, in addition to perceived acceptance, decisions concerning employment made by employers are influenced by other characteristics, for instance, their knowledge about disability, personal beliefs, or attitudes towards PwD.

### 3.4. Evaluation of Acceptance of Types of Disability in Groups of Employees from Poland and Finland

The standardized relationships between measures of attitudes towards four different types of disability in the workplace presented above are below analyzed in detail. Based on the results of research conducted among employers from Poland and Finland, we checked the influence of the place of a company’s operation (country) on the degree of acceptance of people with various types of disability in terms of their possible employment. To this end, we relied on the answers given to two questions from the proprietary questionnaire included in Table A1 in Appendix A, and compared the average results from both countries regarding the acceptance of people with various types of disability by co-workers and the willingness to employ them. Based on the previous CATI research done among employers and co-workers in Poland, in the Polish–Finnish research we applied different scales of responses which were adequate to the content of the questions. Perceived acceptance of PwD was measured on the scale from 1 (lack of acceptance) to 10 (full acceptance), and willingness to employ PwD on the Likert-type scale with four categories from “Strongly No” (1) to “Strongly Yes” (4). The results of the comparisons are presented in Table 10 with *p* value of Mann-Whitney U test.

**Figure 3:**
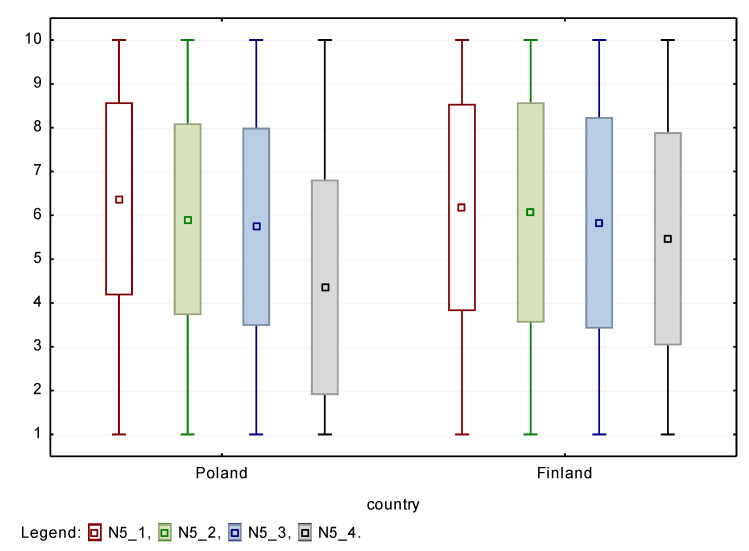
Box-and-whiskers plots for acceptance of four types of PwD.

**Figure 4:**
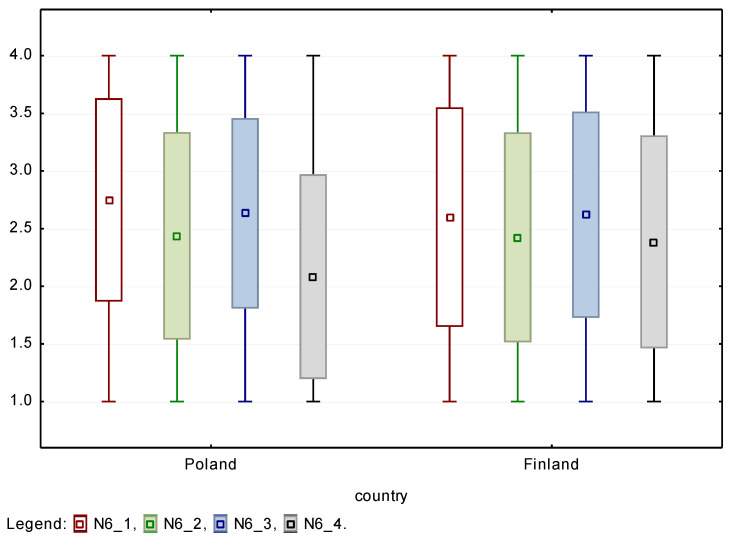
Box-and-whiskers plots for willingness to employ four types of PwD.

In the case of physical and sensory disabilities, no statistically significant differences were found in the opinions of employers from Poland and Finland. Accordingly, these types of disability are likely to be accepted by co-workers at a level comparable in both countries, with the highest values obtained for physical disability. On the other hand, statistically significant differences were found in the acceptance of people with cognitive barriers/difficulties, including intellectual and mental disabilities. Comparing both countries, the acceptance of this type of disability by potential co-workers is much lower in Poland than in Finland. A similar reaction was observed in the case of willingness to employ PwD. Again, no statistically significant differences were noted for physical and sensory disabilities, but a large one to the disadvantage of people with cognitive difficulties regarding possible employment in Poland. Box and whiskers plots are shown for both countries in Figure 3 and Figure 4 with boxes defined as mean ± standard deviation and whiskers from minimum to maximum of the observed responses. The *p* value of nonparametric Friedman ANOVA test for both sets of variables is below 0.001, so differences between assessment of different disability types are statistically significant.

Additionally, the collected data were also used to analyze the dependence cited by many researchers on better attitudes towards PwD displayed by employers who have knowledge in this field or who previously employed people from this group. For this purpose, we performed analogous comparisons of answers to the question on the acceptance and willingness to employ people with various types of disability. In the case of the compared groups of respondents, no significant differences were found for the acceptance of the types of disability by co-workers. However, as far as willingness to employ PwD is concerned, we found statistically significant differences for all types of disability. The respondents who declared good or very good knowledge about disability were more frequently eager to employ people with different types of disability. An analogous reaction was observed among employers representing companies which already hired people from this group. Using the two-way table, we also checked whether there was a significant correlation between the level of knowledge about disability and the employment of people with disabilities. Table 11 presents the size of the respondent groups according to the analysed characteristics. The obtained results indicate a strong correlation between both characteristics confirming a much greater openness towards employing PwD among employers who know the specificity of disability (χ2 = 42.573, *p* value < 0.001).

## 4. Discussion

The confirmatory factor analysis confirmed the acceptance of the measurement model for the entire sample from the CATI research (Polish employers and employees). Both the values of factor structure elements and the model quality measures confirmed the adequacy of the measurement tool. A detailed comparative analysis of the ADS scale in terms of a set of demographic and professional characteristics using modelling and assuming group division was conducted with the use of nested models. We did not observe any significant differences in the models, estimated on the basis of the answers of all respondents, broken down by gender and by the role played in the workplace (employer/employee). In the employers’ sub-sample, it was possible to state the invariance of the model in terms of company’s size, while in relation to the sector, the results are close to significance level. In the sub-sample of employees, we verified invariance in terms of age and no differences in the values of factor loadings were found. The results of multiple-group analysis allow us to conclude that the measurement model is invariant in terms of the analysed demographic and professional characteristics, which indicates the adequacy of the WHOQOL scale as a tool for measuring attitudes towards PwD. This adequacy is also indicated by the works of other authors who used this scale for various studies in different contexts—Power and Green [22] positively evaluated its psychometric properties, Zheng et al. [38] used it to analyze differences in the attitudes of three groups (caregivers, PwD, public) taking into account socio-demographic characteristics, Palad et al. [39] considered a Filipino version of the scale, Bredemeier et al. [40] verified the properties of the Brazilian version, whereas Ma and Hsieh [41] considered it as one of the questionnaires to study stigmatizing attitudes among healthcare students in Taiwan. Interestingly, the results are in contrast to the findings of other authors—Lyon and Houser [42] stated a lack of reliability of the ADS scale for use with nurse educators, and Cantorani et al. [43] pay attention to the fact that the scale has theoretical limitations connected with not taking into account important aspects—accessibility and autonomy.

The attitudes of respondents towards different types of disability were also compared by other researchers who came to a conclusion that openness does not really depend on the type of disability, although they did not show any analysis of correlation between attitudes and types of disability. Guzowski et al. [44] studied the willingness to help people with nine different types of disability. Although there were differences between some types of disability, the authors identified the following as common factors contributing to a more open attitude of respondents: satisfaction with their lives and a high level of empathy. Huskin et al. [45] studied attitudes towards 10 different types of disability and measured, as the main factor of openness, lower social distance scores across all types of contacts with persons with disabilities. This confirms the conclusion that people declaring openness towards PwD are open to various types of disability because of the influence of other personal characteristics on their general openness.

The conducted research has shown that the country in which the company operates differentiates between the degree of acceptance of various types of disability, mainly in the case of cognitive disability, which can be seen in the differences in the answers given by employers from Poland and Finland. This means that important factors shaping the perception of disability in a given community are cultural determinants. The obtained results confirm the previous findings of other authors. For example, research conducted among Israeli and Palestinian students [46] treats the cultural conditioning of the perception of visual impairment, whereas research conducted in different US states [47] deal with the different perception of employees with disabilities.

Unfortunately, the high acceptance of physical disability in Poland also confirms the fact that in post-communist countries disability is still associated with a person moving in a wheelchair, whereas other types of disability are hardly ever noticed. Moreover, public space is full of disability symbols depicting a person in a wheelchair, whereas “accessibility” is mainly perceived in terms of architectural solutions and lifts. This is confirmed by, among others, the results of semiotic and qualitative research [18]. Meanwhile, the need to perceive diverse aspects related to disability has been underlined for years in international reports on this subject [48].

Additionally, it is worth emphasizing that knowledge of disability possessed by employers is an important factor increasing their openness towards PwD which manifests itself in practical action, for example, employing people from this group. It seems that a positive perception of disability is conditioned by knowledge in this area, and the declaration of good or very good knowledge in this field is accompanied by more frequent employment of people with disability. The research results are consistent with the results obtained by other authors [17,49].

## 5. Conclusions

The analyses carried out on representative samples of employers and employees from Poland confirmed that the ADS scale is resistant to respondents’ characteristics. The constraint of the research involves making conclusions based on data from one country. In order to support the thesis about the resistance of the ADS test, it would be worth checking whether similar correlations can be observed in other countries.

The results of the research conducted among Polish and Finnish employers showed that openness and willingness to employ PwD can be reduced to summative scales, which means that it is possible to use an approach independent of the type of disability (high Cronbach’s alpha values for both analysed issues). However, more detailed analyses performed on respondents from the samples of Polish and Finnish employers proved that the use of summative scales causes a loss of important information, and, for example, the omission of significant differences in the perception of cognitive disability in both countries. According to the authors, this problem requires further in-depth research. For this purpose, it seems that it is worthwhile to analyze the differences between individual countries in the context of cultural dimensions that may have a significant influence on the perception of disability both in the social space and workplace. The limitation of the study is that it covers only two countries. It would be worth continuing research in other European countries, including those differing in terms of cultural dimensions or welfare state regimes.

## Figures and Tables

**Table 1:** Sample structure in the CATI research.

Characteristic	Characteristic Categories	Percentage of Respondents (N = 1005)
Whole Sample		
Role	Employer	30.0
	Employee	70.0
Gender	Female	55.1
	Male	44.9
Employers		
Sector	Services	51.8
	Production	34.2
	Trade	14.0
Company’s size	Micro and small companies	49.8
	Medium companies	24.9
	Big companies	25.2
Employees		
Age	18–34 years old	33.7
	35–49 years old	41.9
	50+ years old	24.4

**Table 2:** Sample structure in the CAWI research.

Characteristic	Characteristic Categories	Percentage of Respondents (N = 415)		
		Whole Sample	Poland	Finland
Gender	Female	49.3	60.9	36.5
	Male	50.7	39.1	63.5
Employment of PwD	Employs	31.8	27.4	36.5
	Does not employ	68.2	72.6	63.5
Knowledge about disability	Good or very good	35.1	39.0	31.0
	Average or none	64.9	61.0	69.0

**Table 3:** Four-factor model: parameter estimates and significance.

Latent Variable	ADS Item or Correlation	ML Standardized Factor Loading or Correlation	ADF Standardized Factor Loading or Correlation
1. Inclusion	I1	0.560 ***	0.706 ***
	I2	0.548 ***	0.572 ***
	I3	0.531 ***	0.374 ***
	I4	0.531 ***	0.430 ***
2. Discrimination	D1	0.595 ***	0.618 ***
	D2	0.536 ***	0.577 ***
	D3	0.648 ***	0.650 ***
	D4	0.632 ***	0.678 ***
3. Gains	G1	0.598 ***	0.592 ***
	G2	0.602 ***	0.606 ***
	G3	0.532 ***	0.541 ***
	G4	0.479 ***	0.533 ***
4. Prospects	P1	0.453 ***	0.348 ***
	P2	0.575 ***	0.554 ***
	P3	0.453 ***	0.279 ***
	P4	0.483 ***	0.370 ***
Correlations between factors	r (1,2)	0.503 ***	0.511 ***
	r (1,3)	−0.002	0.093
	r (1,4)	0.506 ***	0.371 ***
	r (2,3)	0.297 ***	0.382 ***
	r (2,4)	0.239 ***	0.144 *
	r (3,4)	−0.008	0.113

* *p* < 0.05, *** *p* < 0.001.

**Table 4:** Characteristics and goodness-of-fit statistics for the four-factor model of ADS scale.

Model Measure	ML Score	ADF Score
Number of parameters	38	38
Chi square	320.123	255.341
d.f.	98	98
p	0.000	0.000
CMIN/DF (minimum discrepancy)	3.267	2.606
RMSEA (root mean square error of approximation)	0.048	0.040
GFI (goodness of fit index)	0.960	0.960
AGFI (adjusted goodness of fit index)	0.945	0.945
CFI (comparative fit index)	0.901	0.813
IFI (incremental fit index)	0.902	0.817

**Table 5:** Chi-square difference tests for nested models of ADS scale.

Variable	Nested Model Type	Chi-Square Difference Test Score	df	*p* Value
Role (whole sample)	Measurement weights	5.501	12	0.939
	Structural covariances	26.104	22	0.247
	Measurement residuals	90.121	38	0.000
Gender (whole sample)	Measurement weights	16.583	12	0.166
	Structural covariances	32.032	22	0.077
	Measurement residuals	76.874	38	0.000
Sector (Employers)	Measurement weights	25.841	12	0.011
	Structural covariances	35.405	22	0.035
	Measurement residuals	78.919	38	0.000
Company’s size (Employers)	Measurement weights	5.879	12	0.922
	Structural covariances	11.282	22	0.970
	Measurement residuals	51.287	38	0.073
Age (Employees)	Measurement weights	36.172	24	0.053
	Structural covariances	70.805	44	0.006
	Measurement residuals	143.954	76	0.000

**Table 6:** Correlation matrix for acceptance of PwD types ^1^.

Variable	N5_1	N5_2	N5_3	N5_4
N5_1	1.000	0.678	0.657	0.530
N5_2	0.678	1.000	0.674	0.628
N5_3	0.657	0.674	1.000	0.582
N5_4	0.530	0.628	0.582	1.000

^1^ Variable content—see Table A1 in Appendix A.

**Table 7:** Correlation matrix for willingness to employ PwD types ^1^.

Variable	N6_1	N6_2	N6_3	N6_4
N6_1	1.000	0.527	0.429	0.384
N6_2	0.527	1.000	0.548	0.599
N6_3	0.429	0.548	1.000	0.573
N6_4	0.384	0.599	0.573	1.000

^1^ Variable content—see Table A1 in Appendix A.

**Table 8:** Factor loadings of joint items sets assessing PwD types.

Variable	Factor 1	Factor 2
N5_1	**0.845**	0.076
N5_2	**0.873**	0.130
N5_3	**0.864**	0.059
N5_4	**0.772**	0.225
N6_1	0.087	**0.705**
N6_2	0.119	**0.840**
N6_3	0.096	**0.797**
N6_4	0.146	**0.802**
Explained variance	2.871	2.560
Share in total variance	0.359	0.320

Bold font marks high loading values greater than 0.7 and assignment to a factor.

**Table 9:** Summary of structural equation model between perceived acceptance of PwD and willingness to employ PwD.

Model Parameter/Measure	Estimate/Score
(Acceptance) --> [N5_1]	0.787 *
(Acceptance) --> [N5_2]	0.855 *
(Acceptance) --> [N5_3]	0.803 *
(Acceptance) --> [N5_4]	0.721 *
(Willingness) --> [N6_1]	0.598 *
(Willingness) --> [N6_2]	0.802 *
(Willingness) --> [N6_3]	0.717 *
(Willingness) --> [N6_4]	0.747 *
(Acceptance) --> (Willingness)	0.323 *
Number of parameters	18
Chi square	121.754
d.f.	19
p	0.000
CMIN/DF (minimum discrepancy)	6.408
RMSEA (root mean square error of approximation)	0.113
GFI (goodness of fit index)	0.933
AGFI (adjusted goodness of fit index)	0.873
CFI (comparative fit index)	0.929
IFI (incremental fit index)	0.930

* *p* < 0.001.

**Table 10:** Results of comparison—acceptance among co-workers and the willingness to employ people with various types of disabilities.

Variable	Mean ± St.dev		U Test *p* Value
	Poland	Finland	
The Acceptance among Co-Workers People with			
mobility or manual barriers/difficulties	6. 38 ± 2.203	6.18 ± 2.361	0.570
visual barriers/difficulties	5.91 ± 2.187	6.07 ± 2.510	0.328
hearing or communication barriers/difficulties	5.74 ± 2.260	5.83 ± 2.410	0.601
cognitive barriers/difficulties	4.36 ± 2.457	5.46 ± 2.431	**0.000**
**The Willingness to Employ People with**			
mobility or manual barriers/difficulties	2.75 ± 0.882	2.60 ± 0.951	0.099
visual barriers/difficulties	2.44 ± 0.899	2.42 ± 0.910	0.843
hearing or communication barriers/difficulties	2.63 ± 0.826	2.62 ± 0.894	0.945
cognitive barriers/difficulties	2.08 ± 0.887	2.39 ± 0.923	**0.001**

Bold font marks *p* value lesser than 0.05.

**Table 11:** Employment of PwD and knowledge about disability among employers from CAWI research.

		Employment of PwD		Total
		Does Not Employ	Employs	
Knowledge about disability	No	213	56	269
	Yes	70	76	146
Total		283	132	415

## Data Availability

The dataset presented in the study is available upon request from the corresponding author.

## Appendix A

**Table A1:** Analysed questions from the proprietary questionnaire.

Question	Response Variants and Coding	
N5. In Your Opinion, to What Extent the Following Types of Disability are Accepted by Co-Workers in the Workplace in Poland/Finland?		
N5_1. People with mobility or manual barriers/difficulties.	Scale from 1 to 10, where 1 means “not accepted at all”, whereas 10 “fully accepted”	
N5_2. People with visual barriers/difficulties.		
N5_3. People with hearing or communication barriers/difficulties.		
N5_4. People with cognitive barriers/difficulties, including intellectual and mental disabilities.		
**N6. How Willing would you be to Hire Employees with the Following Disabilities in Your Company?**		
N6_1. People with mobility or manual barriers/difficulties.	definitely no	1
N6_2. People with visual barriers/difficulties.	rather no	2
N6_3. People with hearing or communication barriers/difficulties.	rather yes	3
N6_4. People with cognitive barriers/difficulties, including intellectual and mental disabilities.	definitely yes	4

## References

[1] European Commission Eurostat Activity limitations due to Health Problems. https://ec.europa.eu/eurostat/en/web/products-eurostat-news/-/ddn-20191128-1.

[2] European Commission Eurostat Self-Perceived Long-Standing Limitations in Usual Activities due to Health Problem by Sex, Age and Labour Status. https://ec.europa.eu/eurostat/databrowser/view/hlth_silc_06/default/table?lang=en.

[3] The World Bank Group Disability Inclusion. https://www.worldbank.org/en/topic/disability.

[4] World Health Organization 10 Facts on Disability. https://www.who.int/features/factfiles/disability/en/.

[5] United Nations Building Back Better: Toward a Disability-Inclusive, Accessible and Sustainable Post COVID-19 World. https://www.un.org/en/observances/day-of-persons-with-disabilities.

[6] United Nations Convention on the Rights of Persons with Disabilities. https://www.un.org/development/desa/disabilities/convention-on-the-rights-of-persons-with-disabilities.html.

[7] United Nations Development Programme Disability Inclusive Development in UNDP: Guidance and Entry Points. https://www.undp.org/content/undp/en/home/librarypage/democratic-governance/human_rights/disability-inclusive-development-in-undp.html.

[8] OECD (2010). Sickness, Disability and Work: Breaking the Barriers. A Synthesis of Findings across OECD Countries.

[9] Bonaccio S., Connelly C.E., Gellatly I.R., Jetha A., Martin Ginis K.A. (2020). The Participation of People with Disabilities in the Workplace Across the Employment Cycle: Employer Concerns and Research Evidence. J. Bus. Psychol..

[10] Bruyère S.M. (2016). Disability and Employer Practices. Research across the Disciplines.

[11] Kaye H.S. (2009). Stuck at the bottom rung: Occupational characteristics of workers with disabilities. JOR.

[12] Khan N., Korac-Kakabadse N., Skouloudis A., Dimopoulos A. (2019). Diversity in the workplace: An overview of disability employment disclosures among UK firms. Corp. Soc. Responsib. Environ. Manag..

[13] Meyer H.-D. (2010). Culture and Disability: Advancing Comparative Research. Comp. Sociol..

[14] Popovich P.M., Scherbaum C.A., Scherbaum K.L., Polinko N. (2003). The assessment of attitudes toward individuals with disabilities in the workplace. J. Psychol..

[15] Palad Y.Y., Barquia R.B., Domingo H.C., Flores C.K., Padilla L.I., Ramel J.M.D. (2016). Scoping review of instruments measuring attitudes toward disability. Disabil. Health J..

[16] Antonak R.F., Livneh H. (2000). Measurement of attitudes towards persons with disabilities. Disabil. Rehabil..

[17] Burke J., Bezyak J., Fraser R.T., Pete J., Ditchman N., Chan F. (2013). Employers’ Attitudes Towards Hiring and Retaining People with Disabilities: A Review of the Literature. Aust. J. Rehabil. Couns..

[18] Kwiatkowska-Ciotucha D., Załuska U., Grześkowiak A. (2020). Osoby z Niepełnosprawnością na Otwartym Rynku Pracy. Bariery Skutecznej Inkluzji w Miejscu Pracy.

[19] Jeffress M.S. (2019). Disability framing in a Caribbean University newspaper. Tout Moun Caribb. J. Cult. Stud..

[20] Chan F., Strauser D., Gervey R., Lee E.-J. (2010). Introduction to demand-side factors related to employment of people with disabilities. J. Occup. Rehabil..

[21] Dunstan D.A., Maceachen E. (2014). A theoretical model of co-worker responses to work reintegration processes. J. Occup. Rehabil..

[22] Power M.J., Green A.M. (2010). The Attitudes to Disability Scale (ADS): Development and psychometric properties. J. Intellect. Disabil. Res..

[23] Power M.J., Green A.M. (2010). Development of the WHOQOL disabilities module. Qual. Life Res..

[24] Power M.J. The WHOQOL-DISABILITIES Module–Manual. https://www.who.int/mental_health/evidence/WHOQOL_DIS_Manual.pdf.

[25] Załuska U., Grześkowiak A., Kozyra C., Kwiatkowska-Ciotucha D. (2020). Measurement of Factors Affecting the Perception of People with Disabilities in the Workplace. Int. J. Environ. Res. Public Health.

[26] Dionne C.D., Gainforth H.L., O’Malley D.A., Latimer-Cheung A.E. (2013). Examining implicit attitudes towards exercisers with a physical disability. Sci. World J..

[27] Brown T.A. (2015). Confirmatory Factor Analysis for Applied Research.

[28] Hoyle R.H. (2015). Handbook of Structural Equation Modeling.

[29] Arbuckle J.L. (2017). IBM^®^ SPSS^®^ Amos™ 25 User’s Guide.

[30] Byrne B.M. (2010). Structural Equation Modeling with AMOS. Basic Concepts, Applications, and Programming.

[31] Ferguson G.A., Takane Y. (2005). Statistical Analysis in Psychology and Education.

[32] Bollen K.A., Long J.S. (1993). Testing Structural Equation Models.

[33] StatSoft, Inc (2014). STATISTICA (Data Analysis Software System), Version 12.

[34] Hair J.F., Black W.C., Babin B.J., Anderson R.E. (2014). Multivariate Data Analysis.

[35] Browne M.W., Cudeck R. (2016). Alternative Ways of Assessing Model Fit. Sociol. Methods Res..

[36] Diamantopoulos A., Siguaw J.A. (2009). Introducing LISREL. A Guide for the Uninitiated.

[37] Weston R., Gore P.A. (2016). A Brief Guide to Structural Equation Modeling. Couns. Psychol..

[38] Zheng Q., Tian Q., Hao C., Gu J., Tao J., Liang Z., Chen X., Fang J., Ruan J., Ai Q. (2016). Comparison of attitudes toward disability and people with disability among caregivers, the public, and people with disability: Findings from a cross-sectional survey. BMC Public Health.

[39] Palad Y., Louie Ignacio M., Genoguin R.K., Ellieza Perez K., Rom Lunar F. (2021). Filipino Attitudes to Disability Scale (Fil-ADS(D)): Factor Structure Validation and an Assessment of Filipino Attitudes. Scand. J. Disabil. Res..

[40] Bredemeier J., Agranonik M., Perez T., Pio M., Fleck M. (2015). Evidence of Validity of the Brazilian Version of ADS: Assessment of Attitudes towards Disabilities. SM J. Community Med..

[41] Ma H.-I., Hsieh C.-E. (2020). Questionnaires on stigmatizing attitudes among healthcare students in Taiwan: Development and validation. BMC Med. Educ..

[42] Lyon L., Houser R. (2016). Psychometric Evaluation of the Attitudes to Disability Scale for Use With Nurse Educators. J. Nurs. Meas..

[43] Cantorani J.R.H., Pedroso B., Vargas L.M., Picinin C.T., Pilatti L.A., Gutierrez G.L. (2019). International and Brazilian Versions of WHOQOL-DIS: (in)adequacy to its Underpinnings. Braz. Arch. Biol. Technol..

[44] Guzowski A., Kułak-Bejda A., Stelcer B., Jasiński M., Łukaszuk C., Cybulski M., Kułak W. (2016). Medical students’ perceptions of people with disabilities. Prog. Health Sci..

[45] Huskin P.R., Reiser-Robbins C., Kwon S. (2017). Attitudes of Undergraduate Students Toward Persons with Disabilities: Exploring Effects of Contact Experience on Social Distance Across Ten Disability Types. Rehabil. Couns. Bull..

[46] Soffer M. (2019). Culture, causal attributions to visual impairments, and stigma: A mediation model. Disabil. Health J..

[47] Gilbride D.D., Stensrud R., Ehlers C., Evans E., Peterson C. (2000). Employers’ attitudes toward hiring persons with disabilities and vocational rehabilitation services. J. Rehabil..

[48] World Health Organization, World Bank (2011). World Report on Disability.

[49] Nelissen P.T.J.H., Hülsheger U.R., van Ruitenbeek G.M.C., Zijlstra F.R.H. (2015). How and when stereotypes relate to inclusive behavior toward people with disabilities. Int. J. Hum. Resour. Manag..

